# Endoscopic Retrograde Cholangiopancreatography (ERCP) in Patients With Liver Cirrhosis: Analysis of Trends and Outcomes From the National Inpatient Sample Database

**DOI:** 10.1097/MCG.0000000000001573

**Published:** 2021-06-09

**Authors:** Shantanu Solanki, Asim Kichloo, Dushyant S. Dahiya, Dhanshree Solanki, Jagmeet Singh, Farah Wani, Michael Albosta, Subash Ghimire, Khwaja F. Haq, Hafiz M.A. Khan, Syed-Mohammed Jafri, Mohammad Arsalan Siddiqui, Tobias Zuchelli

**Affiliations:** * Department of Medicine, Geisinger Commonwealth School of Medicine, Scranton; ¶ Hospitalist Department; ** Department of Gastroenterology, Guthrie Robert Packer Hospital, Sayre, PA; Departments of † Medicine; ∥ Family Medicine, Samaritan Medical Center, Watertown, NY; ‡ Department of Medicine, Central Michigan University College of Medicine, Saginaw; # Department of Gastroenterology, Henry Ford Hospital, Detroit, MI; § Department of Public Health, Rutgers University, New Brunswick, NJ

**Keywords:** gastroenterology, ERCP, cirrhosis, pancreatitis, cholecystitis

## Abstract

We aimed to assess outcomes of patients with liver cirrhosis who underwent therapeutic or diagnostic endoscopic retrograde cholangiopancreatography (ERCP) to determine whether these patients had different outcomes relative to patients without cirrhosis.

**Background:**

ERCP is an important procedure for treatment of biliary and pancreatic disease. However, ERCP is relatively technically difficult to perform when compared with procedures such as esophagogastroduodenoscopy or colonoscopy. Little is known about how ERCP use affects patients with liver cirrhosis.

**Study:**

Using patient records from the National Inpatient Sample (NIS) database, we identified adult patients who underwent ERCP between 2009 and 2014 using International Classification of Disease, Ninth Revision coding and stratified data into 2 groups: patients with liver cirrhosis and those without liver cirrhosis. We compared baseline characteristics and multiple outcomes between groups and compared outcomes of diagnostic versus therapeutic ERCP in patients with cirrhosis. A multivariate regression model was used to estimate the association of cirrhosis with ERCP outcomes.

**Results:**

A total of 1,038,258 hospitalizations of patients who underwent ERCP between 2009 and 2014 were identified, of which 31,294 had cirrhosis and 994,681 did not have cirrhosis. Of the patients with cirrhosis, 21,835 (69.8%) received therapeutic ERCP and 9459 (30.2%) received diagnostic ERCP. Patients with cirrhosis had more ERCP-associated hemorrhages (2.5% vs. 1.2%; *P*<0.0001) compared with noncirrhosis patients but had lower incidence of perforations (0.1% vs. 0.2%; *P*<0.0001) and post-ERCP pancreatitis (8.6% vs. 7%; *P*<0.0001). Cholecystitis was the same between groups (2.3% vs. 2.3%; *P*<0.0001). In patients with cirrhosis, those who received therapeutic ERCP had higher post-ERCP pancreatitis (7.9% vs. 5.1%; *P*<0.0001) and ERCP-associated hemorrhage (2.7% vs. 2.1%; *P*<0.0001) but lower incidences of perforation and cholecystitis (0.1% vs. 0.3%; *P*<0.0001) and cholecystitis (1.9 vs. 3.1%; *P*<0.0001) compared with those who received diagnostic ERCP.

**Conclusions:**

Use of therapeutic ERCP in patients with liver cirrhosis may lead to higher risk of complications such as pancreatitis and postprocedure hemorrhage, whereas diagnostic ERCP may increase the risk of pancreatitis and cholecystitis in patients with cirrhosis. Comorbidities in cirrhosis patients may increase the risk of post-ERCP complications and mortality; therefore, use of ERCP in cirrhosis patients should be carefully considered, and further studies on this patient population are needed.

Endoscopic retrograde cholangiopancreatography (ERCP), since its introduction in 1968, has become a vital endoscopic procedure for managing biliary and pancreatic diseases. Initially introduced as a diagnostic modality in 1968, the first therapeutic ERCP was performed in 1974. Over the years, the clinical demand for ERCP has increased significantly, but usage trends have shown a disproportionate rise of therapeutic ERCPs being performed compared with diagnostic ERCPs. This could be attributed to the development of better imaging modalities for the gastrointestinal tract, but may also be due to the less invasive nature of ERCP, the more dramatic resulting cures for life-threatening conditions, and evidence of improved survival in patients with malignancies such as cholangiocarcinoma.^1^,^2^ Relative to more commonly used standard gastrointestinal procedures such as esophagogastroduodenoscopy and colonoscopy, ERCP is technically far more difficult to perform, requires greater physician skill, and has a longer learning curve to attain high proficiency. These characteristics may contribute to the significant differences in utilization statistics for ERCP within different hospital settings and patient population demographics. ERCP also carries a higher risk of complications, and research has shown that ERCP complications may reach as high as 9.7% with a mortality rate of 0.7% in the general population.^3^–^5^ The most common complications include post-ERCP pancreatitis (PEP), hemorrhage, perforations of the viscera, and post-ERCP cholecystitis (PEC).

Although a large amount of quantitative data is currently available on the utilization trends of ERCP, baseline population characteristics, predictors of complications, and outcomes of ERCP hospitalizations for the general population, as well as outcomes for patients with liver cirrhosis have not been investigated. Thus, we conducted a study using the NIS database to assess patients who underwent ERCP between 2009 and 2014. We sought to identify the utilization trends of therapeutic and diagnostic ERCP in patients with liver cirrhosis and to analyze the association of liver cirrhosis with ERCP complications and outcomes compared with patients without liver cirrhosis.

## MATERIALS AND METHODS

### Data Source

The NIS is a publicly available all-payer health care cost and utilization project (HCUP) database designed to produce US regional and national estimates.^6^,^7^ HCUP is a family of health care databases and related software tools and products released by the Agency for Healthcare Research and Quality (AHRQ). The NIS contains data from >7 million hospital stays per year and approximates a 20% stratified sample of discharges from US community hospitals. HCUP databases are limited data sets.^6^ Under the Health Insurance Portability and Accountability Act (HIPAA), review by an institutional Review Board (IRB) is not required for the use of limited data sets.^8^ Therefore, our study was exempt from IRB review.

### Study Population and Design

We queried the NIS database to identify hospitalizations that occurred between 2009 and 2014 where diagnostic and therapeutic ERCP was a primary or secondary procedural diagnosis. A total of 224,475,443 hospitalizations between 2009 and 2014 were identified in the NIS. We used ICD-9-CM codes depicted in Supplement 1 (Supplemental Digital Content 1, http://links.lww.com/JCG/A750) to determine study groups. All patients below 18 years of age were excluded. We then identified patients with liver cirrhosis by using ICD-9-CM diagnosis codes 571.2, 571.5, and 571.6, which have been used previously.^9^–^11^

For patients with cirrhosis, we compared demographics, hospital-level characteristics (geographical region, size, and teaching status), and patient-level characteristics (insurance and income status) between therapeutic and diagnostic ERCP hospitalizations. See supplement 2 (Supplemental Digital Content 2, http://links.lww.com/JCG/A751) for a description of our data elements. Also, we determined the rates of comorbidities using codes for the Elixhauser comorbidity index provided by HCUP.^12^

### Endpoints

We estimated the association of cirrhosis with outcomes in patients who received ERCP. Primary outcomes were in-hospital mortality, discharge status, and ERCP-related complications (PEP, bowel perforation, cholecystitis, and ERCP-associated hemorrhage). Secondary outcomes were predictors of ERCP-related complications and in-hospital mortality.

### Statistical Analysis

SAS 9.3 (SAS Institute, Cary, NC) was used for all analyses. We used weighted values designated by HCUP to produce nationally representative estimates.^13^ Categorical variables were compared by χ^2^ test and continuous variables were compared using the student *t* test or Wilcoxon rank-sum test. To determine the association of cirrhosis on ERCP outcomes, we composed multivariate logistic regression models after adjusting them for confounding factors. *P*-value <0.05 was considered statistically significant.

## RESULTS

### Trends of ERCP in Patients With Cirrhosis

**FIGURE 1:**
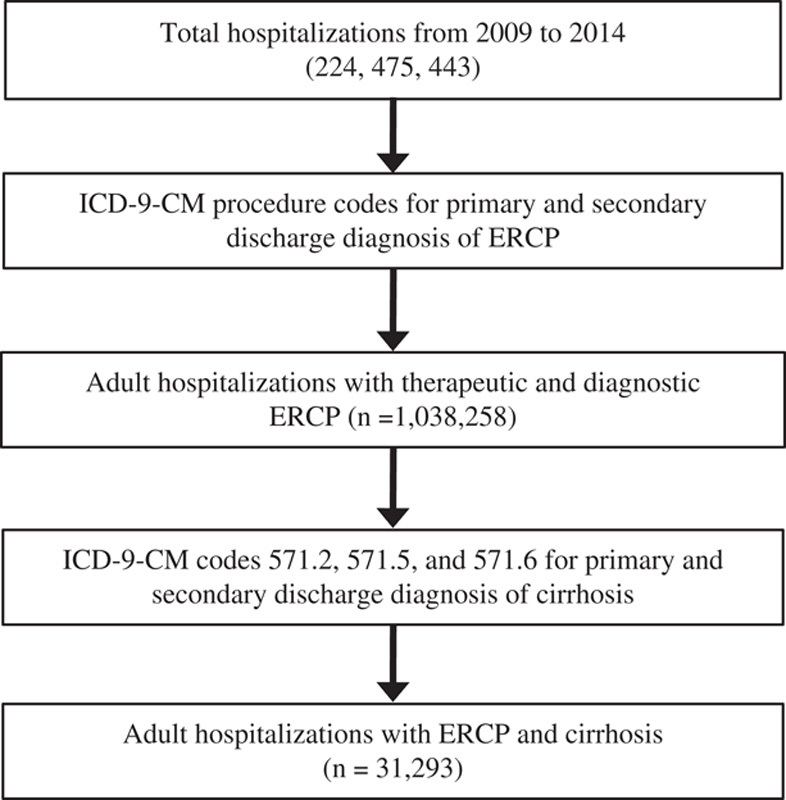
Sequential derivation of study population from the NIS database (2009-2014). ERCP indicates endoscopic retrograde cholangiopancreatography.

**FIGURE 2:**
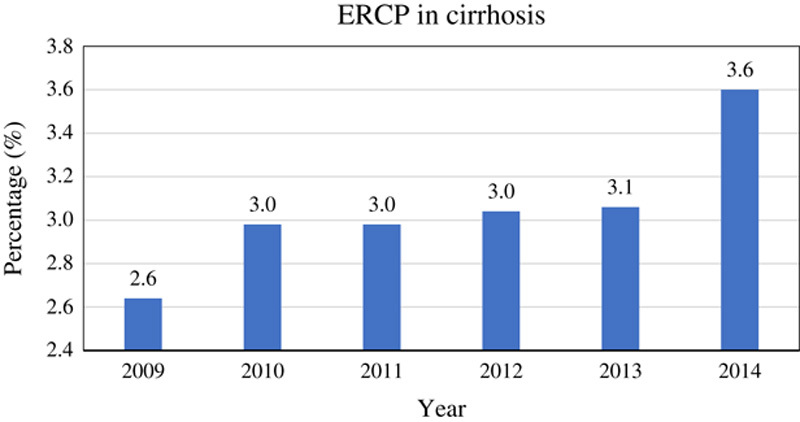
Yearly increase in percentage of ERCP procedures for patients with liver cirrhosis. ERCP indicates endoscopic retrograde cholangiopancreatography.

A total of 1,038,258 adult hospitalizations for patients who received ERCP between 2009 and 2014 were identified in the NIS (Fig. 1), out of which 31,294 (3.05%) had concurrent cirrhosis and 994,681 did not have cirrhosis. Of the patients with cirrhosis, 21,835 (69.8%) received therapeutic and 9459 (30.2%) received diagnostic ERCP (Table 1). A significant increase in the proportion of ERCP hospitalizations with a concurrent diagnosis of cirrhosis was observed from 2.6% in 2009 to 3.6% in 2014 (*P*<0.0001) (Fig. 2).

**TABLE 1:** Number of Therapeutic and Diagnostic Endoscopic Retrograde Cholangiopancreatography (ERCP) Procedures in Patients With Cirrhosis From the National Inpatient Sample Database From 2009 to 2014

Procedure Type	2009	2010	2011	2012	2013	2014	Total (%)
Therapeutic	2651	3538	3690	3630	3820	4505	21,835 (69.8)
Diagnostic	1740	1584	1520	1560	1385	1670	9459 (30.2)
Total ERCP							31,294 (100)

### Baseline Characteristics

For patients with cirrhosis, most ERCPs were performed in those who were in the 45- to 64-year range compared with patients without cirrhosis (51.2% vs. 30%; *P*<0.0001). In addition, most patients with cirrhosis were men (59.7% vs. 40.3%, *P*<0.0001) of Caucasian ethnicity (62.8%) (Table 2). Patients with cirrhosis had fewer ERCPs performed as compared with patients without cirrhosis in small hospitals (8% vs. 10%; *P*<0.0001), and they were more commonly performed for cirrhosis patients in large hospitals (69.9% vs. 65.6%) (Table 2). While fewer patients with cirrhosis received ERCP in rural hospitals (3.1% vs. 5.3%; *P*<0.0001) and in urban nonteaching hospitals (29.6% vs. 38.9%; *P*<0.0001), cirrhosis patients were more likely to receive ERCP in teaching hospitals (67.4% vs. 55.9%; *P*<0.0001) (Table 2). Medicare/Medicaid was the primary insurance for patients with cirrhosis relative to patients without cirrhosis (64.4% vs. 58.2%; *P*<0.0001), whereas private insurance (25.5% vs. 31.3%; *P*<0.0001) or self-pay (10.2% vs. 10.5%; *P*<0.0001) were used less for cirrhosis patients (Table 2). In-hospital mortality was higher for patients with cirrhosis (4.5% vs. 1.4%; *P*<0.0001) (Table 2), and cirrhosis was associated with higher hospital length of stay (LOS) (9 vs. 6 d; *P*<0.0001).

**TABLE 2:** Baseline Characteristics of Patients With and Without Cirrhosis From the NIS Database (2009-2014)

Characteristics	Without Cirrhosis (n=994,681)	With Cirrhosis (n=31,294)	Overall	*P*
Age in years (%)				<0.0001
18-44	25.0	10.1	24.6	
45-64	30.0	51.2	30.6	
65-84	35.0	34.8	35.0	
≥85	10.0	3.9	9.8	
Sex (%)				<0.0001
Male	39.7	59.7	40.3	
Female	60.3	40.3	59.7	
Race (%)				<0.0001
White	67.8	62.8	67.7	
Black	9.1	10.4	9.2	
Hispanic	15.6	18.3	15.7	
Others	7.5	8.5	7.5	
Hospital bed size (%)				<0.0001
Small	10.0	8.0	9.9	
Medium	24.4	22.1	24.4	
Large	65.6	69.9	65.7	
Hospital region (%)				<0.0001
Northeast	19.4	18.2	19.4	
Midwest	22.1	21.7	22.1	
South	35.3	33.9	35.3	
West	23.2	26.2	23.3	
Hospital type (%)				<0.0001
Rural	5.3	3.1	5.2	
Urban nonteaching	38.9	29.6	38.6	
Teaching	55.9	67.4	56.2	
Primary insurance (%)				<0.0001
Medicare/Medicaid	58.2	64.4	58.4	
Private including HMO	31.3	25.5	31.1	
Uninsured/self-pay	10.5	10.2	10.5	
Type of admission (%)				<0.0001
Emergent or urgent	93.0	92.6	93.0	
Elective	7.0	7.4	7.0	
Disposition status (%)				<0.0001
Home	86.7	78.5	86.4	
Facility	11.9	17.0	12.1	
Died	1.4	4.5	1.5	

### Association of Cirrhosis With ERCP Outcomes

More patients with cirrhosis experienced ERCP-associated hemorrhage compared with patients without cirrhosis (2.5% vs. 1.2%; *P*<0.0001); however, cirrhosis patients had lower rates of ERCP-associated bowel perforation (0.1% vs. 0.2%; *P*<0.0001) and PEP (7% vs. 8.6%; *P*<0.0001) compared with noncirrhosis patients. Cholecystitis was the same between the 2 groups at 2.3% (*P*<0.0001) (Table 3).

**TABLE 3:** Endoscopic Retrograde Cholangiopancreatography (ERCP)-related Complications in Patients With and Without Cirrhosis From the NIS Database (2009-2014)

Complications	Without Cirrhosis (%) (n=994,681)	With Cirrhosis (%) (n=31,294)	*P*
Post-ERCP pancreatitis	8.6	7.0	<0.0001
Perforation	0.2	0.1	<0.0001
Cholecystitis	2.3	2.3	<0.0001
ERCP-associated hemorrhage	1.2	2.5	0.001

For patients with cirrhosis, those who received therapeutic ERCP had higher rates of PEP (7.9% vs. 5.1%; *P*<0.0001) and ERCP-associated hemorrhage (2.7% vs. 2.1%; *P*<0.0001) (Table 4), but interestingly lower rates of perforation (0.1% vs. 0.3%; *P*<0.0001) and cholecystitis (1.9% vs. 3.1%; *P*<0.0001) compared with those who received diagnostic ERCP.

**TABLE 4:** Complications Related to Therapeutic Endoscopic Retrograde Cholangiopancreatography (ERCP) Versus Diagnostic ERCP in Patients With Cirrhosis in the NIS Database (2009-2014)

Complications	Therapeutic ERCP (%)	Diagnostic ERCP (%)	*P*
Post-ERCP pancreatitis	7.9	5.1	<0.0001
Perforation	0.1	0.3	<0.0001
Cholecystitis	1.9	3.1	<0.0001
ERCP-associated hemorrhage	2.7	2.1	0.001

### Predictors of In-Hospital Complications and Mortality in Hospitalized Cirrhosis Patients Who Received ERCP

On multivariable-adjusted analysis, with every 1-point increase in the Elixhauser comorbidity index in patients with cirrhosis, the risk of ERCP-associated complications increased significantly [odds ratio (OR), 1.11; 95% confidence interval (CI), 1.07-1.16; *P*<0.0001] (Table 5). However, age, hospital size, and bed size were not found to have any significant association with complications (Table 5) in these patients. Also, patients with cirrhosis who developed any ERCP-associated complication showed a higher risk of mortality (OR, 1.48; 95% CI, 1.03-2.14; *P*=0.04) relative to those who did not develop any complications (Table 6). Age older than 85 years was associated with the highest risk of mortality for cirrhosis patients (OR, 2.76; 95% CI, 1.29-5.91; *P*=0.01) (Table 6). Small (OR, 0.52; 95% CI, 0.28-0.95; *P*=0.03) and medium (OR, 0.71; 95% CI, 0.52-0.98; *P*=0.04) sized hospitals showed lower risk of mortality for cirrhosis patients when compared to large hospitals. With each 1-point increase in Elixhauser comorbidity index, the risk of mortality increased significantly for hospitalized cirrhosis patients who received ERCP (OR, 1.19; 95% CI, 1.13-1.26; *P*<0.0001) (Table 6).

**TABLE 5:** Multivariate Analysis of Predictors of Endoscopic Retrograde Cholangiopancreatography–related Complications in Patients With Cirrhosis

Procedure Type	Odds Ratio (95% Confidence Interval)	*P*
Elixhauser index (every 1-point increase)	1.11 (1.07-1.16)	<0.0001
Age in years		
18-44	Referent	
45-64	1.06 (0.79-1.43)	0.69
65-84	0.89 (0.65-1.22)	0.46
≥85	0.80 (0.48-1.34)	0.40
Gender		
Male	Referent	
Female	1.11 (0.94-1.31)	0.21
Race		
White	Referent	
Black	0.89 (0.68-1.16)	0.39
Hispanic	0.93 (0.74-1.18)	0.56
Others	0.99 (0.73-1.35)	0.97
Hospital bed size		
Small	0.75 (0.53-1.06)	0.1
Medium	0.94 (0.76-1.17)	0.60
Large	Referent	
Hospital region		
Northeast	Referent	
Midwest	1.05 (0.80-1.38)	0.73
South	0.93 (0.72-1.20)	0.58
West	0.99 (0.76-1.28)	0.92
Hospital type (%)		
Rural	0.99 (0.62-1.59)	0.98
Urban nonteaching	0.83 (0.68-1.00)	0.05
Teaching	Referent	
Primary insurance (%)		
Medicare/Medicaid	Referent	
Private including HMO	1.18 (0.98-1.43)	0.09
Uninsured/self-pay	0.87 (0.64-1.19)	0.38

**TABLE 6:** Multivariate Analysis of Predictors of Mortality for Endoscopic Retrograde Cholangiopancreatography in Patients With Cirrhosis

Procedure Type	Odds Ratio (95% Confidence Interval)	*P*
Elixhauser index (every 1-point increase)	1.19 (1.13-1.26)	<0.0001
Any complication	1.48 (1.03-2.14)	0.04
Age in years		
18-44	Referent	
45-64	1.87 (1.03-3.38)	0.04
65-84	2.01 (1.09-3.71)	0.03
≥85	2.76 (1.29-5.91)	0.01
Sex		
Male	Referent	
Female	0.89 (0.68-1.17)	0.40
Race		
White	Referent	
Black	1.20 (0.80-1.81)	0.38
Hispanic	0.88 (0.63-1.25)	0.48
Others	1.41 (0.94-2.13)	0.10
Hospital bed size		
Small	0.52 (0.28-0.95)	0.03
Medium	0.71 (0.52-0.98)	0.04
Large	Referent	
Hospital region		
Northeast	Referent	
Midwest	0.66 (0.44-1.00)	0.05
South	0.70 (0.50-1.00)	0.05
West	0.77 (0.53-1.10)	0.15
Hospital type (%)		
Rural	0.87 (0.46-1.68)	0.68
Urban nonteaching	1.10 (0.82-1.46)	0.54
Teaching	Referent	
Primary insurance (%)		
Medicare/Medicaid	Referent	
Private including HMO	1.01 (0.73-1.41)	0.94
Uninsured/self-pay	0.98 (0.59-1.61)	0.92

Furthermore, another multivariate regression analysis was performed for 21,835 patients with cirrhosis who underwent therapeutic ERCP. Although we noted a significant increase in the risk of therapeutic ERCP-associated complications (OR, 1.12; 95% CI, 1.07-1.17; *P*<0.0001) with every 1-point increase in the Elixhauser comorbidity index (Table 7), the patient age, sex, and race did not have a significant association with these complications. In addition, the risk of mortality increased significantly for age older than 85 years and every 1-point increase in the Elixhauser comorbidity index (OR, 1.20; 95% CI, 1.12-1.28; *P*<0.0001) (Table 8).

**TABLE 7:** Multivariate Analysis of Predictors of Therapeutic Endoscopic Retrograde Cholangiopancreatography–related Complications in Patients With Cirrhosis

Procedure Type	Odds Ratio	95% Confidence Interval		*P*
Elixhauser index (every 1-point increase)	1.12	1.07	1.17	<0.0001
Age in years				
18-44		Referent	
45-64	1.05	0.74	1.50	0.79
65-84	0.86	0.59	1.26	0.44
≥85	0.63	0.34	1.19	0.16
Gender				
Male		Referent	
Female	1.07	0.88	1.31	0.49
Race				
White		Referent	
Black	0.84	0.59	1.19	0.32
Hispanic	0.89	0.66	1.19	0.42
Others	1.01	0.71	1.44	0.94
Hospital bed size				
Small	0.60	0.40	0.92	0.02
Medium	0.91	0.70	1.19	0.51
Large		Referent	
Hospital region				
Northeast		Referent	
Midwest	1.07	0.77	1.49	0.69
South	1.02	0.75	1.37	0.92
West	1.00	0.74	1.35	0.99
Hospital type (%)				
Rural	0.68	0.39	1.21	0.19
Urban nonteaching	0.76	0.61	0.95	0.02
Teaching		Referent	
Primary insurance (%)				
Medicare/Medicaid		Referent	
Private including HMO	1.14	0.90	1.43	0.28
Uninsured/self-pay	0.92	0.64	1.32	0.64

**TABLE 8:** Multivariate Analysis of Predictors of Mortality for Therapeutic Endoscopic Retrograde Cholangiopancreatography in Patients With Cirrhosis

Procedure Type	Odds Ratio	95% Confidence Interval		*P*
Elixhauser index (every 1-point increase)	1.20	1.12	1.28	<0.0001
Any complication	1.44	0.91	2.27	0.12
Age in years				
18-44	Referent			
45-64	1.9	0.9	4.1	0.12
65-84	1.9	0.9	4.4	0.11
≥85	3.1	1.2	8.1	0.02
Gender				
Male	Referent			
Female	0.80	0.57	1.12	0.19
Race				
White	Referent			
Black	1.27	0.76	2.13	0.36
Hispanic	0.88	0.56	1.38	0.57
Others	1.46	0.91	2.35	0.12
Hospital bed size				
Small	0.70	0.35	1.40	0.31
Medium	0.68	0.46	1.00	0.05
Large	Referent			
Hospital region				
Northeast	Referent			
Midwest	0.58	0.35	0.98	0.04
South	0.69	0.45	1.05	0.08
West	0.70	0.44	1.10	0.12
Hospital type (%)				
Rural	1.19	0.62	2.28	0.60
Urban nonteaching	1.21	0.87	1.70	0.26
Teaching	Referent			
Primary insurance (%)				
Medicare/Medicaid	Referent			
Private including HMO	0.96	0.64	1.43	0.83
Uninsured/self-pay	0.81	0.42	1.56	0.52

## DISCUSSION

ERCP has been a prominent technological innovation in the field of gastrointestinal endoscopy since its inception in 1968.^14^ ERCP, a combined endoscopic and fluoroscopic procedure, is a more complicated integral therapeutic modality among endoscopic techniques, and it is a highly specialized procedure used for the treatment of pancreatic and biliary system disorders.^15^ Because of its complex nature, ERCP requires specialized equipment and a long learning curve for physicians to develop high proficiency. During the procedure, a specialized side-viewing duodenoscope is guided into the duodenum, allowing instrumentation of the bile and pancreatic ducts. A contrast medium is then injected into the bile and/or pancreatic duct allowing for their radiographic visualization and thereby permitting necessary diagnostic or therapeutic interventions.

Over the past decade, ERCP has shifted from being a diagnostic to a therapeutic modality, which may be due to advances in therapeutic technology, including noninvasive radiologic imaging modalities, sphincterotomes for sphincterotomy, inflatable balloons or stents to dilate strictures, electrocautery to stem hemobilia, and baskets or inflatable balloons to retrieve choledocholithiasis.^16^ Therapeutic ERCP is usually less invasive than surgical interventions, produces dramatic cures for life-threatening conditions, and often improves survival in patients with malignancies such as cholangiocarcinoma. Thus, the popularity of ERCP has increased over other more invasive interventions such as surgery.^1^,^2^ ERCP is superior to traditional interventions in terms of limiting potential trauma to internal structures, simplifying the operation, and reducing recovery times in the diagnosis and treatment of duodenal and pancreatobiliary disorders; however, like any intervention, it carries its own set of complications.^15^

Some common indications for the utilization of ERCP include the following: obstructive jaundice, biliary or pancreatic ductal system disease treatment or tissue sampling, possible pancreatic cancers, pancreatitis of unknown cause, manometry for sphincter of Oddi dysfunction, nasobiliary drainage, biliary stenting for strictures and leakage, drainage of pancreatic pseudocysts, balloon dilation of the duodenal papilla and ductal strictures, sphincterotomy for sphincter of Oddi dysfunction or stenosis, difficulty with biliary stenting, difficulty in accessing pancreatic duct, biliary strictures, or bile duct stones, bile sump syndrome following choledochoduodenostomy and choledochocele, and sphincterotomy in poor surgical candidates with ampullary carcinoma (https://www.asge.org/home/guidelines#biliary-and-pancreatic-endoscopy).

Robust growth in the use of ERCP has been observed in the United States and worldwide. A 10-year population-based cohort study by Coelho-Prabhu et al^17^ in the United States reported that the average implementation of ERCP was 83.1 ERCPs/100,000 persons per year, with an increase from 58 to 104.8 ERCPs/100,000 persons per year over time. Recent reports have shown a significant decrease in diagnostic ERCP procedures by 57% and a concomitant increase in therapeutic procedures by 37%.^18^ This is mainly attributed to an improvement in the noninvasive imaging modalities such as computed tomography, magnetic resonance imaging, and magnetic resonance cholangiopancreatography, along with the increased use of endoscopic ultrasound, due to its relative safety and superiority over ERCP for tissue acquisition.^19^–^22^ Our study focused on the use of ERCP in a specific population that included only those with liver cirrhosis undergoing therapeutic or diagnostic ERCP. The NIS contained 1,038,258 adult hospitalizations with ERCP use between 2009 and 2014 (Fig. 1), out of which 31,294 (3.05%) patients had a concurrent diagnosis of liver cirrhosis. In this subset of liver cirrhosis patients, 21,835 (69.8%) underwent therapeutic ERCP and 9459 (30.2%) underwent diagnostic ERCP (Table 1). The utilizations of diagnostic ERCP in these patients may be to rule out possible etiologies commonly associated with liver cirrhosis such as cholelithiasis, choledocholithiasis, intrahepatic cholangiocarcinoma particularly in patients with primary biliary cholangitis or primary sclerosing cholangitis, pancreatitis, or pancreatic carcinoma. Further analysis of the data revealed that there was a significant increase in the proportion of ERCP hospitalizations with a concurrent diagnosis of cirrhosis from 2.6% in 2009 to 3.6% in 2014 (*P*<0.0001) (Fig. 2), reflecting a rise in the use of ERCP in cirrhotic patients.

Utilization trends for ERCP differ significantly in the general population versus the population with liver cirrhosis. Studies have reported a mean age of 59 ± 19 years with a higher number of women (61%) and white patients (57%) within the general inpatient population where ERCP was used. These trends are followed by an increasing occurrence of ERCP use in the Hispanic population.^23^ The increasing utilization trends of the procedure in the Hispanic population may be explained by the rising prevalence of biliary and pancreatic diseases in this subset population.^24^ In contrast, current studies assessing the utilization of ERCP in patients with liver cirrhosis are limited. Studies that have looked at the utilization trends of ERCP in liver cirrhosis have reported a mean age of 59.26 years predominantly in men (59%).^25^ In patients with liver cirrhosis, our study reported a higher utilization of this procedure in the 45 to 64 years age group (51.2%), predominantly in men (59.7% vs. 40.3%, *P* <0.0001), and more prevalently used for white patients (62.8%), which aligns with previous reports.

Furthermore, we investigated the employment of ERCP in patients with or without liver cirrhosis with respect to different hospital settings and population demographics to identify areas of maximum procedural burden. Our analysis showed that more ERCPs were performed in patients without cirrhosis than in patients with cirrhosis in small- and medium-sized hospitals, whereas more ERCPs were performed in patients with cirrhosis in large hospitals. We believe that these trends are due to the increased referrals of patients with liver cirrhosis from small and medium sized hospitals to larger academic centers due to significant concerns about morbidity and mortality. We also observed that more ERCPs were performed in patients without cirrhosis in rural and urban nonteaching hospitals, whereas more ERCPs were performed in patients with cirrhosis in teaching hospitals. This could be attributed in part to the increased referral of cirrhosis patients for ERCP to large teaching hospitals, a higher concentration of large teaching hospitals in urban or metropolitan areas, and a greater availability of teaching faculty, advanced endoscopists and in-training gastroenterology physicians equipped to manage critical cases. However, it is worth noting that small- and medium-sized hospitals showed a lower risk of mortality for cirrhosis patients compared with large hospitals, which could be explained by a greater referral of critical cases to larger tertiary hospitals. These statistics are of particular importance, as they reflect the need for more advanced endoscopists in rural and suburban settings, along with better equipped hospitals to manage critical cases. With respect to the payment method used for the procedure, we observed that Medicare/Medicaid was the primary insurance used by patients with cirrhosis relative to noncirrhosis patients; however, private insurance and self-pay were the primary health care payment methods for patients without cirrhosis.

Like any intervention, ERCP can lead to complications requiring unplanned admission or prolonged LOS.^26^,^27^ The complications of ERCP can be subdivided into multiple categories based on the site of the injury, timing since the procedure, and the severity of the injury. Table 9 summarizes a complete list of complications of ERCP.^28^ On multivariable-adjusted analysis, we observed that with every 1-point increase in Elixhauser comorbidity index, the risk of total ERCP-associated complications increased significantly. Similarly, from a purely therapeutic ERCP perspective, the risk of therapeutic ERCP-associated complications increased significantly with every 1-point increase in Elixhauser comorbidity index. Here, we will outline the major complications associated with ERCP, compare the significant differences in these complications between the cirrhosis and noncirrhosis patients, and quantify the association of liver cirrhosis on the outcomes of the procedure.

1. Hemorrhage: bleeding is one of the common complications associated with ERCP, and postsphincterotomy bleeding has been reported in up to 2% of ERCP cases.^29^ Immediate bleeding can usually be seen in up to 30% of patients, whereas delayed bleeding can occur up to 2 weeks after the procedure.^30^ Risk factors that increase the risk of postsphincterotomy bleeding include liver cirrhosis, dilated common bile ducts, periampullary diverticulum, precut sphincterotomy, and common bile duct stones.^31^ Interestingly, use of aspirin or nonsteroidal anti-inflammatory drugs have not been shown to increase risk of bleeding.^30^ In our study, ERCP-associated hemorrhage was higher in cirrhosis patients compared with noncirrhosis patients. Furthermore, in the patients with cirrhosis, we noted a higher prevalence of ERCP-associated hemorrhage in those who received therapeutic ERCP versus the diagnostic ERCP. The mainstay of treatment for immediate postsphincterotomy bleeding includes endoscopic therapy using epinephrine injection and/or sprays, hemostatic clips, balloon tamponade, and/or covered stent placement. In addition to making sure that necessary equipment is readily available, it is also important to identify these patients at high risk of bleeding, so that appropriate endoscopy and resuscitation protocols can be activated.
2. Pancreatitis: asymptomatic elevation of serum lipase is a common occurrence after ERCP and may be seen in up to 75% of the patients in the general population, regardless of symptoms.^32^ PEP, the most common complication of ERCP, is defined as new-onset or worsening abdominal pain with elevation of serum amylase (3 or more times the upper limit of reference range) at 24 hours postprocedure and the need for >2 days of pancreatitis-related hospitalization.^33^ The exact pathophysiological mechanism of PEP is poorly understood, but possible mechanisms include the mechanical obstruction of the papillae or the sphincter by instrumentation, hydrostatic injury from the injection of contrast, water, and chemicals, or allergic injury from contrast injection.^34^ The common end-point is the activation of the inflammatory pathway leading to inflammation-mediated damage to the pancreas. Studies have shown that the overall incidence of PEP may be as high as 9.7% with a mortality rate of 0.7%.^35^ However, studies of PEP in patients with liver cirrhosis are limited; hence, our study was designed to investigate this relationship. We showed that the rate of PEP was found to be higher in patients without cirrhosis compared with those with cirrhosis. Because of coding limitations, it is likely that the cirrhosis cohort included patients with liver transplant who were on immunosuppressive medications. Corticosteroids and tacrolimus are noted to have a protective role in the development of PEP in liver transplant recipients.^36^,^37^ This could have been the reason for the lower rate of PEP in patients with cirrhosis. In the population of cirrhosis patients, we observed PEP to be higher after therapeutic ERCP compared with diagnostic ERCP. However, reports have shown that postprocedural pharmacotherapy has not been found to be effective for PEP despite promising results in animal models. Therefore, gastroenterologists have focused their efforts on the prevention of PEP. Various strategies for prevention have been employed, such as careful selection of patients, administration of intraprocedural intravenous fluids, guidewire assisted cannulations, prophylactic pancreatic duct stenting, single dose rectal indomethacin in high risk patients, and consideration of alternative treatment modalities if possible.
3. Perforations: perforation is one of the most feared complications of ERCP and endoscopic sphincterotomy. In current studies, the incidence of ERCP-associated perforations has been shown to be low at 0.39%, but the overall associated mortality was found to be as high as 7.8% of all perforations.^38^ In the general population, endoscopic sphincterotomy was found to be responsible for 41% of the perforations, followed by insertion and manipulation of the endoscope at 26%, and the use of guidewires at 15%.^38^ Although studies do reflect that liver cirrhosis has a negative effect on the overall prognosis of patients with a perforated viscus, there is a dearth of objective data backing this claim. Our study showed that perforations occurred more in the noncirrhotic patients compared with patients with liver cirrhosis (0.2% vs. 0.1%; *P*<0.0001), which may be attributed to a higher number of ERCPs in noncirrhosis patients. We also noted that perforations were lower in cirrhosis patients who received therapeutic ERCP compared with those who received diagnostic ERCP. Higher operator vigilance during therapeutic ERCPs than diagnostic ERCPs could be a reason behind this finding. The diagnosis of ERCP-associated perforations can usually be established during the procedure with the help of the endoscopic view or fluoroscopy. Treatment options can include endoscopic clips, endoscopic purse-string suture, band ligation, covered self-expanding metal stents, endoscopic vacuum therapy or surgical intervention, and depends on the mechanism of the injury, site of injury, degree of the leak and the patient’s condition.^39^ Early diagnosis and treatment play a vital role in the decreasing the overall mortality.
4. Cholecystitis: PEC has gained less traction over the years due to a limited incidence relative to other more common complications of ERCP. Objective data for PEC have mainly been generated in single center retrospective studies that have analyzed incidence and risk factors of PEP. The most recent single center retrospective study published in 2020 by Ting et al^40^ found a PEC incidence of 0.95% in the general population undergoing ERCP. Studies have also reported a slightly higher incidence (1.35%) of PEC within the first 2 weeks after ERCP.^41^ The risk factors that significantly increase the incidence for PEC include the following: a history of chronic cholecystitis, previous acute pancreatitis, gall bladder opacification, biliary metallic stent placement, and a high leukocyte count before the procedure.^41^ Our study mainly focused on investigating the association of liver cirrhosis on PEC. We discovered a similar rate of cholecystitis in patients regardless of cirrhosis status, despite a greater number of ERCPs having been performed in noncirrhosis patients. We also found a higher rate of cholecystitis in cirrhosis patients who underwent diagnostic ERCP compared with those who underwent therapeutic ERCP. This could likely be due to delayed contrast drainage from lack of sphincterotomy. Although PEC is an uncommon complication of ERCP, early detection and intervention is necessary to prevent sequelae such as purulent cholecystitis. Prophylactic measures become essential for patients undergoing ERCP who have risk factors. A significant knowledge gap exists in this realm, with the availability of data only from small single center retrospective studies; hence, we strongly advocate for large multi-center prospective studies to investigate PEC for identifying additional risk factors in patients with or without liver cirrhosis undergoing ERCP.
5. In-hospital mortality and LOS: although patients undergoing ERCP generally have multiple comorbidities, it has been well established that ERCP is itself an independent predictor of in-hospital mortality after controlling for age, COPD, type II diabetes, cirrhosis, and coronary artery disease.^42^ The LOS associated with ERCP is 8.1 days in the general population.^42^ Our study analyzed the association of liver cirrhosis on ERCP outcomes. We found in-hospital ERCP-related mortality to be higher in patients with cirrhosis compared those without cirrhosis. In patients who developed ERCP-associated complications, there was a higher risk of mortality compared with those without complications (Table 6). Age was also linked to a higher mortality rates, and we observed that an age older than 85 years was associated with the highest risk of mortality among all age groups. The results showed that with each 1-point increase in Elixhauser comorbidity index, the risk of mortality increased significantly. From a purely therapeutic ERCP perspective, age older than 85 years and every 1-point increase in Elixhauser comorbidity index was associated with higher risk of mortality. Liver cirrhosis was also associated with a longer LOS compared with the patients without cirrhosis (9 vs. 6 d).

**TABLE 9:** Complications of Endoscopic Retrograde Cholangiopancreatography

Site of Injury	Timing	Severity of Injury
Focal: site of endoscopic contact, that is hemorrhage, perforation and pancreatitis	Immediate: during the procedure	(1) Based on length of hospital stay:
	Early: within the recovery period	Mild: ≤3 nights.
Nonspecific: sites not traversed by the endoscope, that is cardiopulmonary complications	Delayed: focal—within 30 d Nonspecific – first symptom within 3 d	Moderate: 4-10 nights.
	Late: after months or years	Severe: >10 nights, ICU admissions, or surgery
		Fatal: death within 30 d due to the procedure or longer if under continued in-patient treatment.
		(2) Based on other indicators of severity:
		Need for blood transfusion Need for additional interventions Total length of stay Permanent residual disability

ERCP continues to be an invaluable technique for therapeutic interventions in pancreatic and biliary disorders. It differs greatly compared with other endoscopic procedures in its predominantly therapeutic intent, necessity for typical performance in hospitals, its steep learning curve, and trend of occasionally causing severe complications.^16^ Our study showed that patients with liver cirrhosis undergoing ERCP have a higher rate of morbidity and mortality compared with the general population. Hence, as the rates of utilization of ERCP and liver cirrhosis continue to increase worldwide, we suggest that large multicentric prospective studies are required to determine the impact of liver cirrhosis on ERCP outcomes and to put forward recommendations for ERCP in patients with liver cirrhosis.

## CONCLUSIONS

Our study corroborated the shift in trends of ERCP from a diagnostic to a therapeutic modality. We observed a higher rate of ERCP-associated hemorrhage in patients with cirrhosis compared with patients without cirrhosis, whereas ERCP-related perforations and PEP were higher in patients without cirrhosis. Occurrence of PEC was similar in cirrhosis and patients without cirrhosis. Of the patients with cirrhosis, ERCP-associated hemorrhage and PEP were higher in those who received therapeutic ERCP, but perforations and PEC were higher in those who received diagnostic ERCP. Comorbidities in patients with cirrhosis may increase the risk of post-ERCP complications and mortality; therefore, use of ERCP in patients with cirrhosis should be carefully considered, and further studies on this patient population are needed.

## Supplementary Material

SUPPLEMENTARY MATERIAL

Supplemental Digital Content is available for this article. Direct URL citations appear in the printed text and are provided in the HTML and PDF versions of this article on the journal's website, www.jcge.com.
