# Supplementary material for: Endoscopic Retrograde Cholangiopancreatography (ERCP) in Patients With Liver Cirrhosis: Analysis of Trends and Outcomes From the National Inpatient Sample Database
Source: J Clin Gastroenterol. 2021 Jun 9;56(7):618–26. doi: 10.1097/MCG.0000000000001573 (PMC9257052; doi:10.1097/MCG.0000000000001573)
Supplement: SUPPLEMENTARY MATERIAL [file mcg-56-618-s001.docx]

**Supplement 1: Description of ICD-9 codes**

| **Codes** | **Description** |
| --- | --- |
| **Cirrhosis** | |
| 571.2 | Alcoholic cirrhosis |
| 571.5 | Cirrhosis from all others other than alcohol |
| 571.6 | Biliary cirrhosis |
| **Diagnostic ERCP** | |
| 51.10 | Endoscopic retrograde cholangiopancreatography (ERCP) |
| 51.11 | Endoscopic retrograde cholangiography (ERC) |
| 51.14 | Other closed (endoscopic) biopsy of biliary duct or sphincter of Oddi |
| 52.13 | Endoscopic retrograde pancreatography (ERP) |
| 52.14 | Closed (endoscopic) biopsy of pancreatic duct |
| **Therapeutic ERCP** | |
| 51.84 | Endoscopic dilation of ampulla and biliary duct |
| 51.85 | Endoscopic sphincterotomy and papillotomy |
| 51.86 | Endoscopic insertion of naso-biliary drainage tube |
| 51.87 | Endoscopic insertion of stent (tube) into bile duct |
| 51.88 | Endoscopic removal of stone(s) from biliary tract |
| 52.93 | Endoscopic insertion of stent (tube) into pancreatic duct |
| 52.94 | Endoscopic removal of stone(s) from pancreatic duct |
| 52.97 | Endoscopic insertion of naso-pancreatic drainage tube |
| 52.98 | Endoscopic dilation of pancreatic duct |
