# Supplementary material for: Endoscopic Retrograde Cholangiopancreatography (ERCP) in Patients With Liver Cirrhosis: Analysis of Trends and Outcomes From the National Inpatient Sample Database
Source: J Clin Gastroenterol. 2021 Jun 9;56(7):618–26. doi: 10.1097/MCG.0000000000001573 (PMC9257052; doi:10.1097/MCG.0000000000001573)
Supplement: SUPPLEMENTARY MATERIAL [file mcg-56-618-s002.docx]

**Supplement 2: Description of data elements**

| **Data element** | **Description** |
| --- | --- |
| **Location** |  |
| Rural | A non-metropolitan statistical area |
| Urban | A metropolitan statistical area |
| Teaching | A hospital with an AMA-approved residency program or have membership in the Council of Teaching Hospitals |
| **Median Household Income** |  |
| Quartile 1 | $1-24,999 |
| Quartile 2 | $25,000-34,999 |
| Quartile 3 | $35,000-44,999 |
| Quartile 4 | $45,000 or more |

| **Bed-size categories** | | | |
| --- | --- | --- | --- |
| ***Location and Teaching Status*** | **Hospital Bed-size** | | |
|  | **Small** | **Medium** | **Large** |
| **Northeast region** | | | |
| Rural | 1-49 | 50-99 | 100+ |
| Urban, nonteaching | 1-124 | 125-199 | 200+ |
| Urban, teaching | 1-249 | 250-424 | 425+ |
| **Midwest region** | | | |
| Rural | 1-29 | 30-49 | 50+ |
| Urban, nonteaching | 1-74 | 75-174 | 175+ |
| Urban, teaching | 1-249 | 250-374 | 375+ |
| **Southern region** | | | |
| Rural | 1-39 | 40-74 | 75+ |
| Urban, nonteaching | 1-99 | 100-199 | 200+ |
| Urban, teaching | 1-249 | 250-449 | 450+ |
| **Western region** | | | |
| Rural | 1-24 | 25-44 | 45+ |
| Urban, nonteaching | 1-99 | 100-174 | 175+ |
| Urban, teaching | 1-199 | 200-324 | 325+ |
